# Difficulty in differentiating between IgG4-related hepatic inflammatory pseudotumor and intrahepatic cholangiocarcinoma

**DOI:** 10.1007/s12328-020-01245-x

**Published:** 2020-10-09

**Authors:** Ai Hamano, Reiko Yamada, Kazunari Kurata, Junya Tsuboi, Hiroyuki Inoue, Kyosuke Tanaka, Noriyuki Horiki, Yoshiyuki Takei

**Affiliations:** 1 Department of Gastroenterology and Hepatology, Mie University Graduate School of Medicine, 2-174 Edobashi, Tsu, Mie 514-8507 Japan; 2 Department of Endoscopy, Mie University Graduate School of Medicine, Tsu, Japan

**Keywords:** IgG4-related disease, Hepatic inflammatory pseudotumor, Intrahepatic cholangiocarcinoma, Liver biopsy

## Abstract

A 71-year-old man on prednisolone for immunoglobulin (Ig) G4-related renal disease showed increased carbohydrate antigen (CA) 19–9 level; abdominal enhanced computed tomography (CT) showed a lesion in the left lateral segment and dilatation of the peripheral biliary duct. He was referred to our hospital for detailed examination for suspected intrahepatic cholangiocarcinoma. CT and magnetic resonance imaging findings were similar to those for intrahepatic cholangiocarcinoma. However, endoscopic retrograde cholangiopancreatography showed a smooth narrowing of the bile duct which suggested inflammatory disease. Liver biopsy was performed; IgG4-related hepatic inflammatory pseudotumor (IPT) was diagnosed. IgG4-related hepatic IPTs are rare diseases that develop in association with the development of sclerosing cholangitis. Most of these lesions develop in the hepatic hilum and the imaging findings of these tumors are similar to those of hilar cholangiocarcinomas. Thus, hepatic IPTs are difficult to differentiate from malignancy; in some cases, surgical resection has been considered for establishing the diagnosis. In the present case, we could diagnose hepatic IPT on the basis of liver biopsy, which is the recommended approach in cases of suspected hepatic IPT.

## Introduction

Immunoglobulin (Ig) G4-related disease is a well-known disorder, which can affect various organs of the body, such as the pancreas, biliary tree, liver, kidneys, salivary glands, breast, pericardium, skin, lungs, meninges, and the pituitary gland [1]. IgG4-related disease is characterized by abundant IgG4-positive plasma cell infiltration and high serum IgG4 levels. The disease sometimes manifests as tumorous lesions, and the association between IgG4-related disease and inflammatory pseudotumors (IPTs) has been suggested. The Organizing Committee, comprised of 35 IgG4-related disease experts, recommended that “IgG4-related hepatopathy” was a preferred nomenclature instead of hepatic IPT [2].

IgG4-related hepatic IPTs develop in association with development of sclerosing cholangitis [3, 4]. IgG4-related hepatic IPTs are rare disease entities, and it is difficult to distinguish between these IPTs and malignant tumors, such as hilar cholangiocarcinoma or intrahepatic cholangiocarcinoma (periductal infiltrating type). Here, we describe a case of IgG4-related hepatic IPT, diagnosed on the basis of liver biopsy without surgical resection, in which it was difficult to distinguish between IgG4-related hepatic IPT and periductal infiltrating type intrahepatic cholangiocarcinoma.

## Case report

A 71-year-old man was referred to our hospital for detailed examination of a lesion in the liver. He had a history of smoking for 51 years (20 cigarettes per day), no history of alcohol consumption, and no remarkable family history. He had received treatment for IgG4-related renal disease and lymphocytic hypophysitis. He had been taking 4 mg prednisolone daily and 60 μg desmopressin acetate hydrate every second day. IgG4-related renal disease had been well controlled, and IgG4 level had been around 180 mg/dL (5–117 mg/dL). Although he was asymptomatic, his carbohydrate antigen (CA) 19–9 level increased (from 32.4 U/ml to 304.9 U/ml), and abdominal enhanced computed tomography (CT) showed a lesion in the left lateral segment and dilatation of the peripheral biliary duct.

The laboratory data showed elevated levels of hepatobiliary enzymes [aspartate transaminase (AST) 40 IU/l (normal range; 13–40 IU/l), alkaline phosphatase (ALP) 612 IU/l (106–322 IU/l), γ-glutamyl transpeptidase (GTP) 92 IU/l (13–64 IU/l)] and tumor markers [carcinoembryonic antigen (CEA) 8.4 ng/ml (− 5.2 ng/ml), CA19-9 304.9 U/ml (− 36.8 U/ml)]. IgG4 was also elevated; 205 mg/dL. The other parameters were within the normal ranges.

**Fig. 1:**
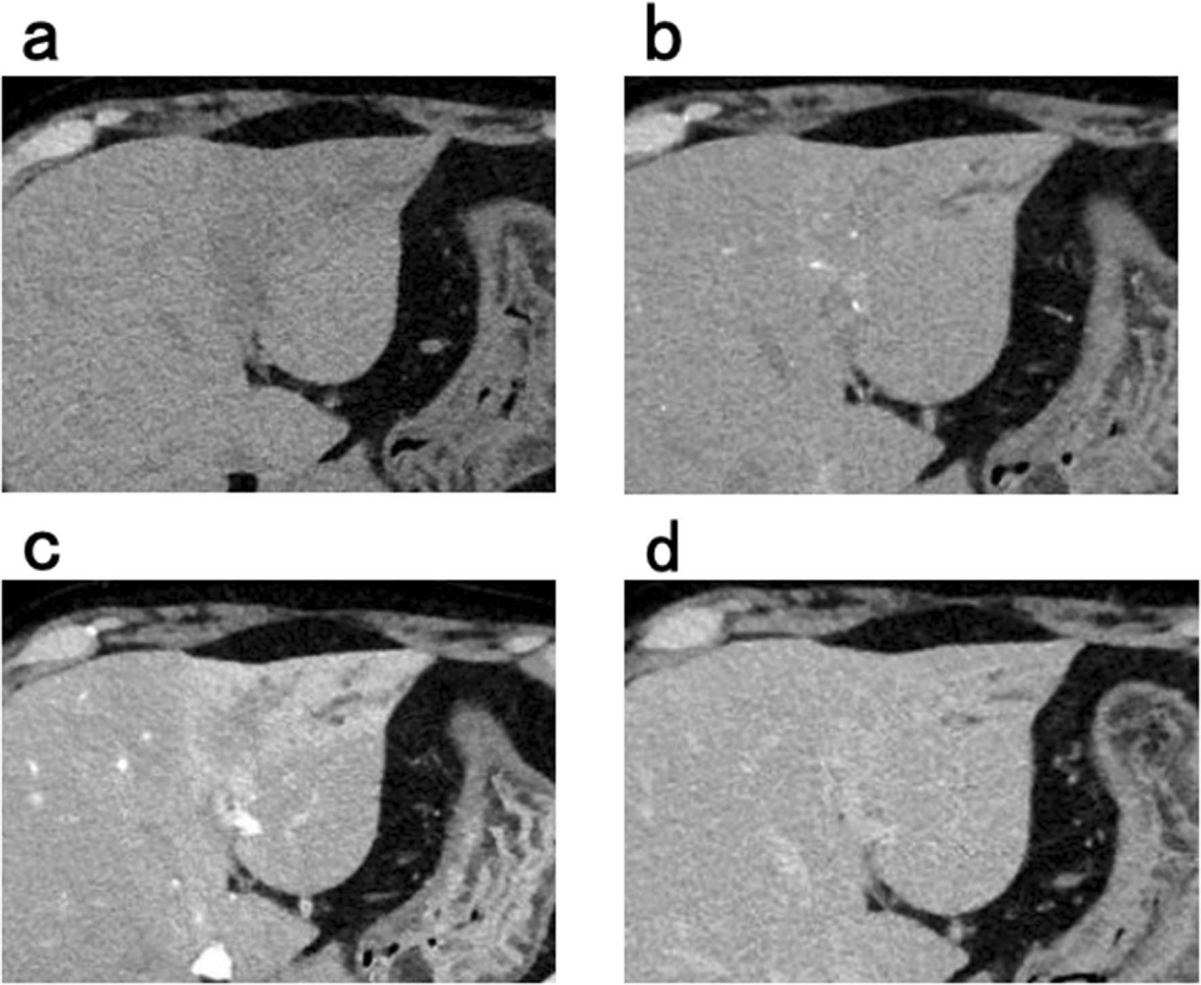
Abdominal enhanced computed tomography image showing **a** demonstrated an ambiguous lesion in S3 of the liver and dilatation of the intrahepatic and peripheral bile ducts. The lesion was poorly enhanced on **b** early and **c** portal phase, and **d** showed delayed enhancement on late phase

**Fig. 2:**
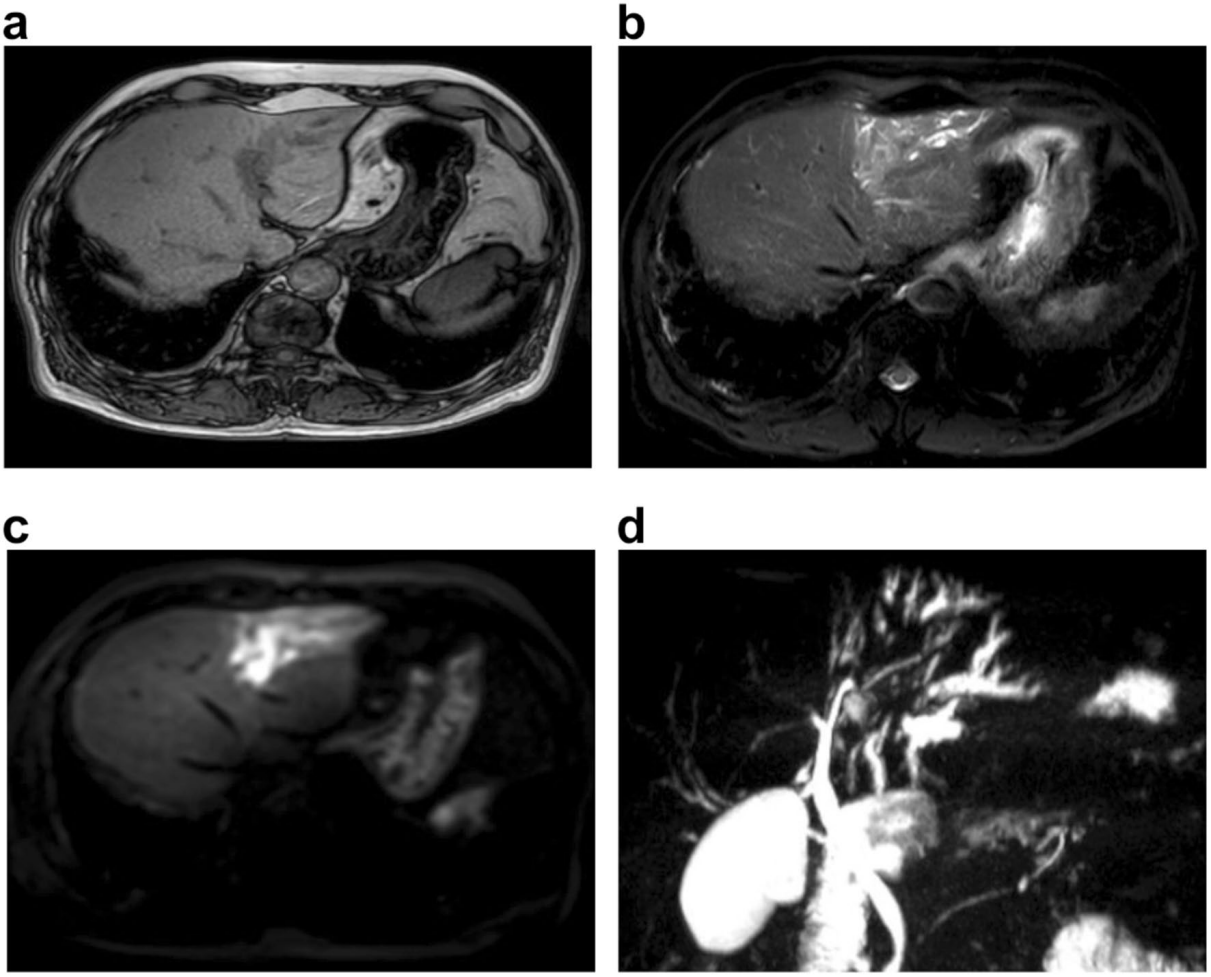
Magnetic resonance imaging scans showing **a** a low-intensity mass on a T1-weighted image, **b** a faint high-intensity mass on a T2-weighted image, **a** and a high-intensity mass with a low apparent diffusion coefficient value on DWI (**c**). Magnetic resonance cholangiopancreatography (MRCP) image showing dilatation of the peripheral bile duct

**Fig. 3:**
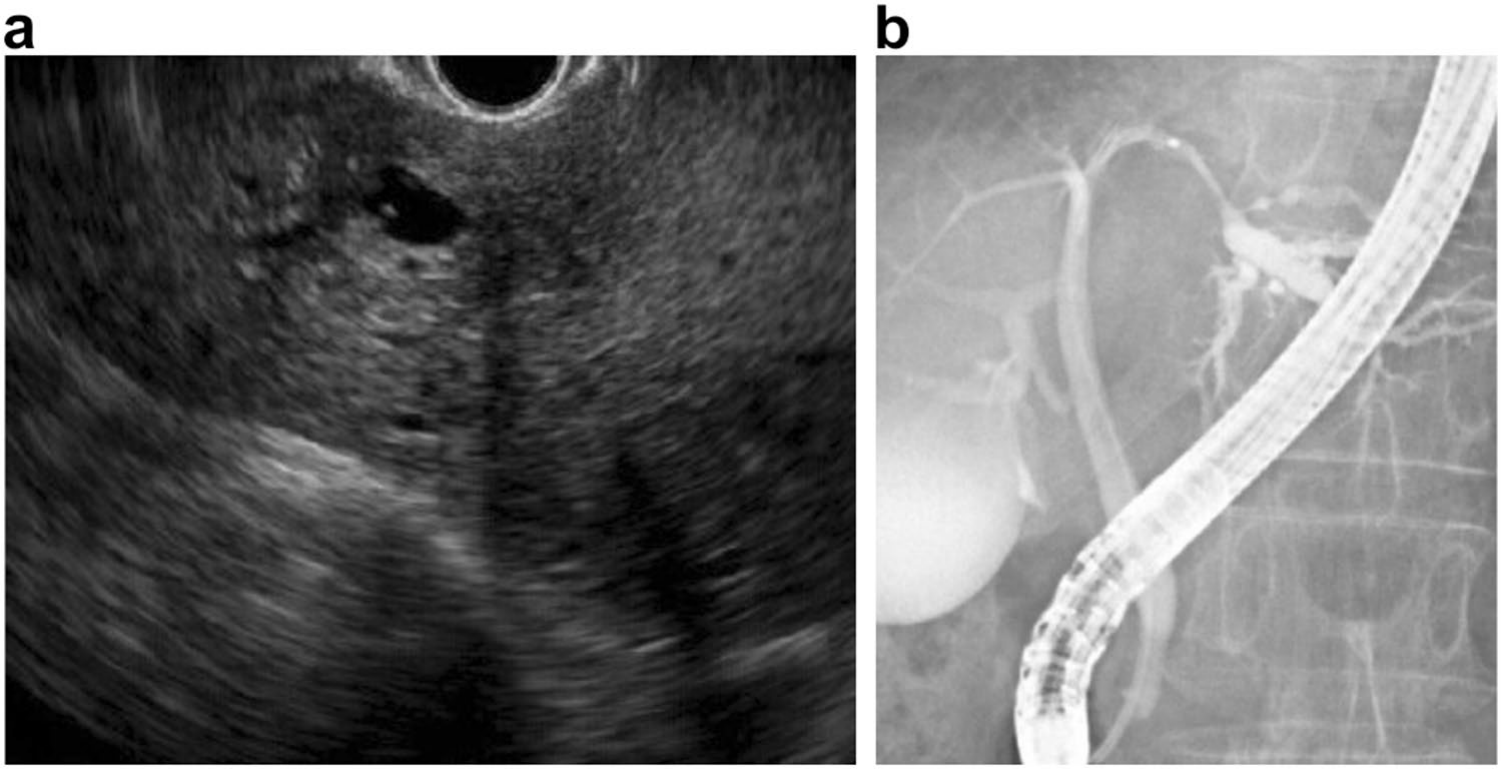
**a** Endoscopic ultrasound (EUS) image showing dilatation of B3 and a poorly marginated hypoechoic area. **b** Endoscopic retrograde cholangiopancreatography (ERCP) image showing smooth narrowing of the bile duct in B3 and a partially beaded appearance of B3

Abdominal enhanced CT revealed ambiguous lesion in segment 3 (S3) of the liver, and dilatation of the intrahepatic and peripheral bile duct. The lesion was poorly enhanced on early and portal phase, and showed delayed enhancement on late phase (Fig. 1a–d). Abdominal magnetic resonance imaging (MRI) revealed a low-intensity lesion on a T1-weighted image in S3 of the liver (Fig. 2a). This lesion appeared as a faint high-intensity poorly marginated mass on a T2-weighted image (Fig. 2b) and a high-intensity mass on diffusion-weighted images with low apparent diffusion coefficient (ADC) values (Fig. 2c). Magnetic resonance cholangiopancreatography (MRCP) showed dilatation of the peripheral bile duct (Fig. 2d). Endoscopic ultrasound (EUS) showed a poorly circumscribed hypoechoic area in S3 and dilatation of bile duct 3 (B3) (Fig. 3a).

**Fig. 4:**
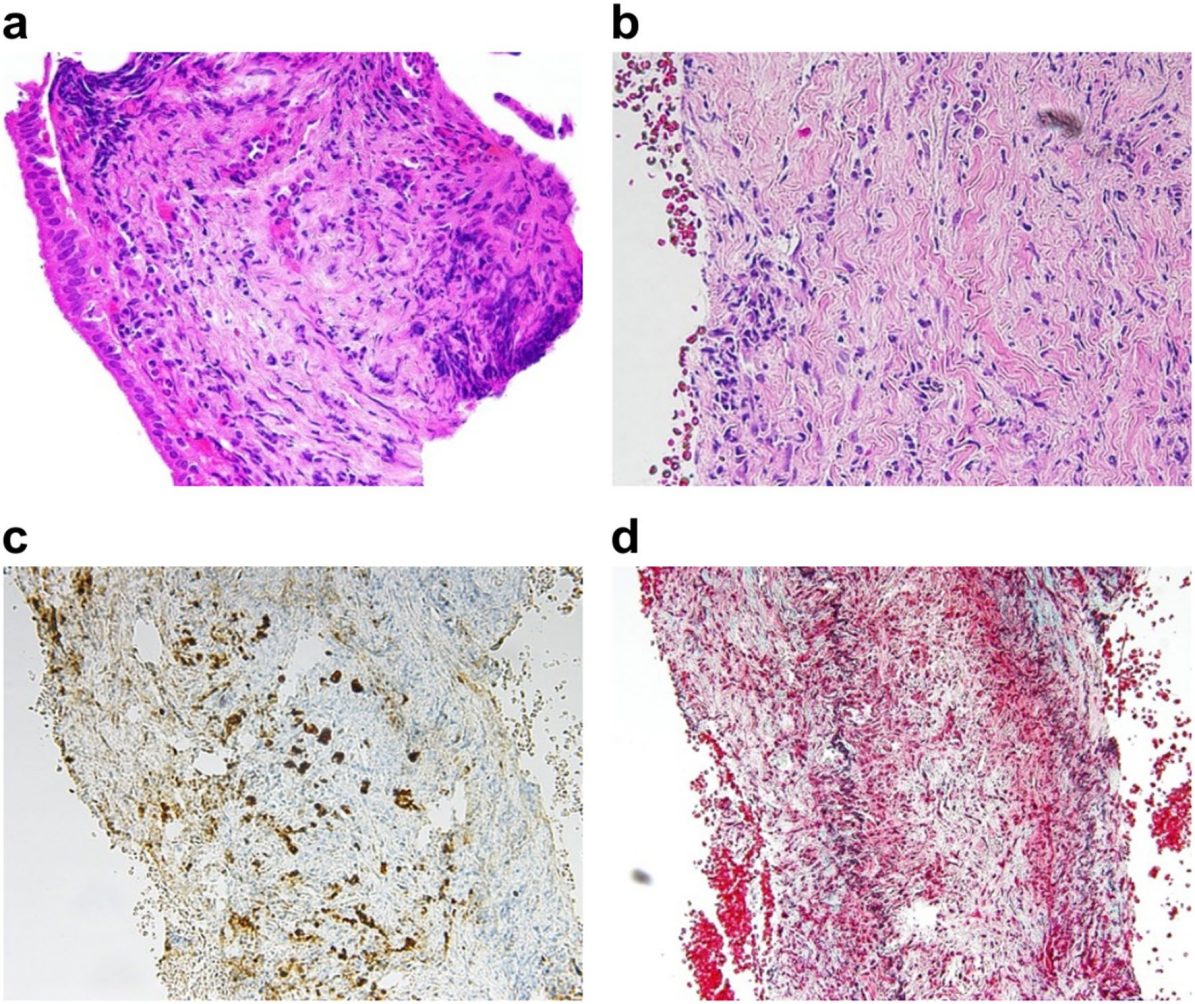
**a** Bile duct biopsy image showing absence of atypical cells. **b**–**d**: Pathological analysis of hepatic inflammatory pseudotumors. **b** Inflammatory pseudotumors show inflammatory cell infiltration and fibrosis. **c** Inflammatory pseudotumors show IgG4-positive cell infiltration. **d** Obliterative phlebitis is observed.

Endoscopic retrograde cholangiopancreatography (ERCP) showed smooth narrowing of the bile duct in B3, and a partially beaded appearance of B3 (Fig. 3b). Bile duct biopsy (Fig. 4a) and brush cytology of the bile ducts did not show atypical cells.

**Fig. 5:**
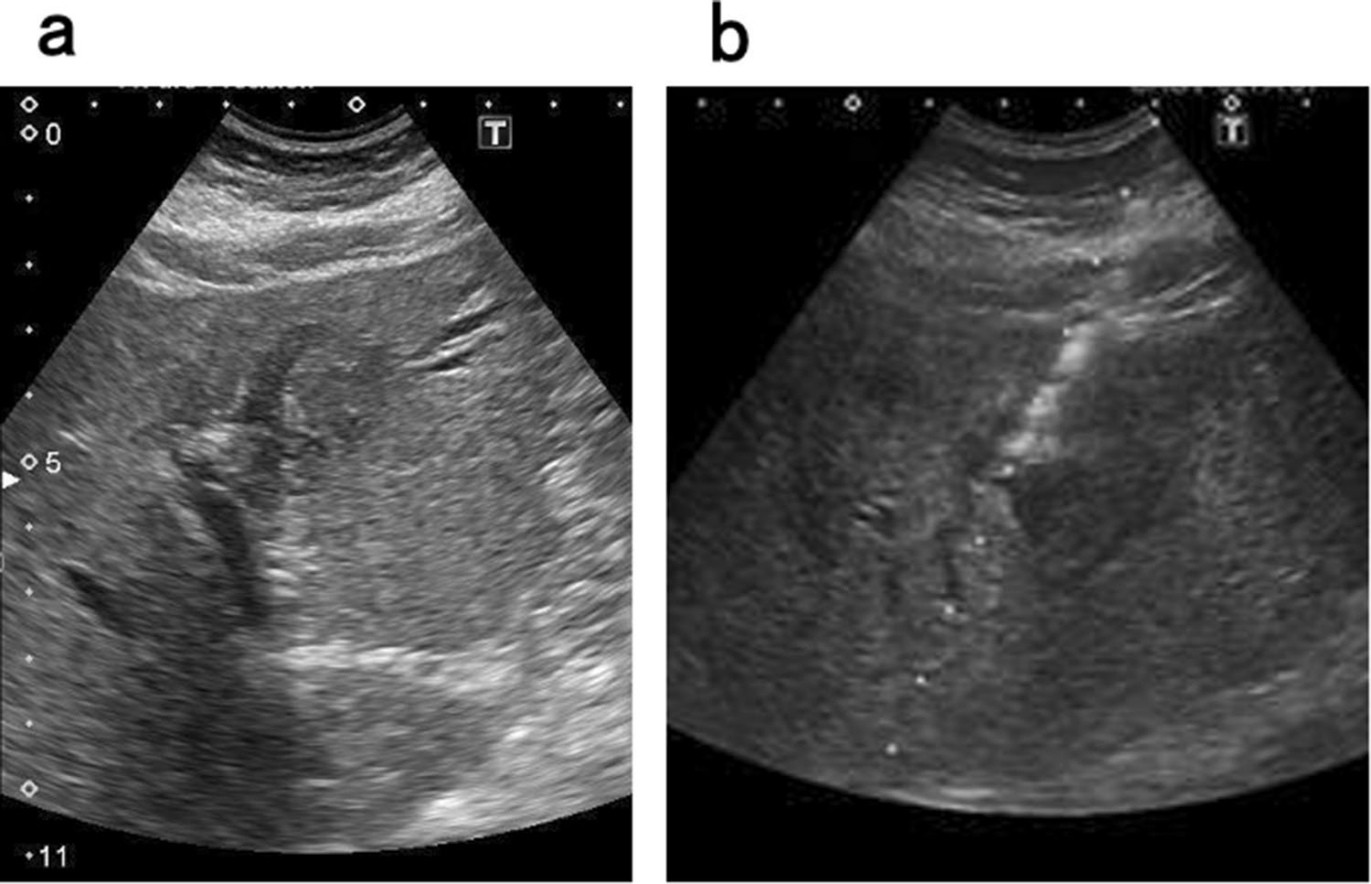
**a** Echo image showing a poorly marginated hypoechoic area in S3. **b** Liver biopsy was performed for the lesion in S3 using 19G needle

At first, we suspected intrahepatic cholangiocarcinoma (periductal infiltrating type) based on the CT and MRI findings. However, inflammatory disease, like IgG4-related hepatic IPT, was also suspected because ERCP showed smooth narrowing of the bile duct, bile duct biopsy/cytology did not show cellular atypia, and the patient’s medical history included IgG4-related disease. Because the biopsy specimen was very small for IgG4 staining, percutaneous liver biopsy was performed for the lesion in S3 using 19G needle (Fig. 5a, b). The pathological findings of liver biopsy were fibrosis with inflammatory cell infiltration (Fig. 4b), IgG4-positive cell infiltration (20 per hpf; Fig. 4c), and obliterative phlebitis (Fig. 4d). Hence, IgG4-related hepatic IPT was diagnosed.

**Fig. 6:**
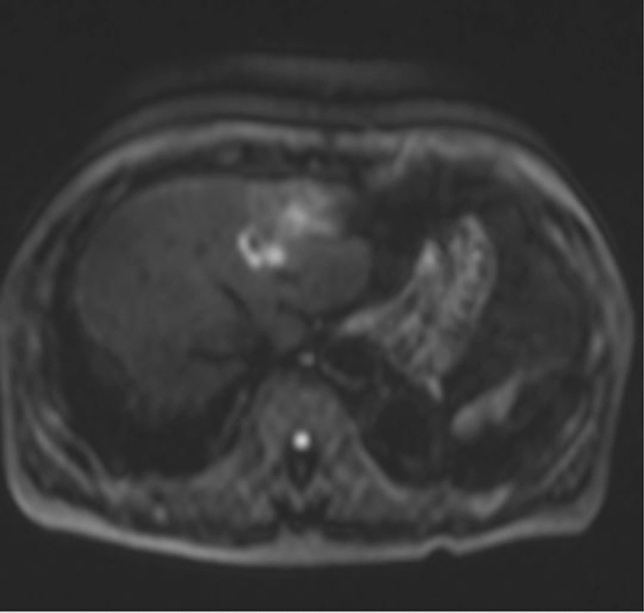
Two months later, MRI showed the size of the mass in S3 reduced on DWI

After diagnosis, the patient was administered 10 mg prednisolone daily i.e., the dosage was increased from 4 to 10 mg. Two months later, the serum IgG4 level had improved from 205 mg/dL to 137 mg/dL. The laboratory data of liver function slightly improved. MRI showed a decrease in the size of the mass in S3 and an improvement in inflammation (Fig. 6). One year later, the serum IgG4 level had further improved to 73 mg/dL.

## Discussion

Hepatic IPT is pathologically classified into two types, namely, fibrohistiocytic and lymphoplasmacytic IPT [3]. Each characteristic of hepatic IPTs, fibrohistiocytic and lymphoplasmacytic type, is shown in Table 1. Zen et al. characterized fibrohistiocytic IPTs by xanthogranulomatous inflammation, multinucleated giant cells, and neutrophilic infiltration. These tumors mostly developed in the peripheral hepatic parenchyma as mass-forming lesions. The fibrohistiocytic type IPTs manifest as subjective symptoms, such as fever, abdominal pain, and general malaise. In contrast, lymphoplasmacytic IPTs show diffuse lymphoplasmacytic infiltration and prominent eosinophilic infiltration. A significantly greater number of IgG4-positive plasma cells are observed in the lymphoplasmacytic IPTs than fibrohistiocytic IPTs. Liver dysfunction was incidentally detected in patients with lymphoplasmacytic IPTs on routine laboratory testing. These tumors usually develop in the hepatic hilum and are distributed along the hilar bile ducts. The lymphoplasmacytic IPTs are similar to hilar cholangiocarcinomas or intrahepatic cholangiocarcinomas (periductal infiltrating type).

**Table 1:** Clinical characteristics comparison of hepatic inflammatory pseudotumors between fibrohistiocytic and lymphoplasmacytic types

	Fibrohistiocytic type	Lymphoplasmacytic type
Sex	No sex difference	Male > Female
Hepatic lobes	Left lobe = Right lobe	Left lobe > Right lobe
Location	Peripheral liver	Hilar bile ducts
Shapes	Mass-forming type	Periductal infiltrating type
Clinical presentation	Subjective symptoms (fever, abdominal pain, general malaise)	Liver dysfunction by laboratory test
Histological features	Xanthogranulomatous inflammation Multinucleated giant cells Nodular eosinophilic deposition Venous occlusion Inflammatory cholangitis	Eosinophil infiltration Plasma cell infiltration Russell bodies Obliterative phlebitis Sclerosing cholangitis

IgG4-related hepatic IPTs are rare diseases that develop in association with the development of sclerosing cholangitis [4]. Most of these lesions develop in the hepatic hilum and the imaging findings of these tumors are similar to those of hilar cholangiocarcinomas. IPT is considered a complication of other diseases, such as autoimmune pancreatitis and sclerosing cholangitis [5]. The pathological characteristics are storiform fibrosis, obliterative phlebitis, eosinophilic infiltration, diffuse infiltration with IgG4-positive cells or plasma cells, and IgG4-/IgG-positive plasma cell ratios > 40% [6, 7]. IgG4-related hepatic IPTs are asymptomatic and are sometimes detected concomitantly with other conditions, such as liver dysfunction. Prednisolone is efficacious [7, 8] and is associated with better disease prognosis.

It is difficult to differentiate between IgG4-related hepatic IPTs and cholangiocarcinomas, because the imaging findings were similar for the conditions; in some cases, surgical resection was considered for establishing the diagnosis [5]. Histological confirmation on biopsies and accurate diagnosis are important for avoiding the unnecessary administration of treatment, such as surgical resection, to patients with IgG4-related hepatic IPTs [9, 10]. Chougule et al. mentioned that the IgG4-related IPTs diagnosed on biopsies with requisite features showed prompt response to steroids indicating specificity of histopathological findings in predicting treatment response [11]. Thus, to confirm histologically using biopsy sample is important for treatment.

In the present case, we could diagnose hepatic IPT on the basis of liver biopsy, which is the recommended approach in cases of suspected hepatic IPT [12]. Although we initially suspected intrahepatic cholangiocarcinoma (periductal infiltrating type) based on the enhanced CT and MRI findings, ERCP showed smooth narrowing of the bile duct, similar to that noted in inflammatory disease. Furthermore, bile duct biopsy and brush cytology findings of the stenotic bile ducts did not show cellular atypia. Hence, we suggest that it is important to include IgG4-related hepatic IPT in the differential diagnosis of liver lesions observed on imaging analysis.
